# Supplementary material for: Unbiased human genomic characterization of polyglutamine disorder genes to guide biological understanding and therapeutic strategies
Source: HGG Adv. 2025 Nov 17;7(1):100547. doi: 10.1016/j.xhgg.2025.100547 (PMC12719159; doi:10.1016/j.xhgg.2025.100547)
Supplement: Table S1. PolyQ gene-trait GWAS pairs retained at different L2G thresholds [file mmc1.pdf]

HGG A, Volume 7

Supplemental information

Unbiased human genomic characterization of polyglutamine disease genes to guide biological understanding and therapeutic strategies

Kevin Lucy Namuli, Britt I. Drögemöller, and Galen E.B. Wright

Table S1. Influence of L2G threshold on number of polyQ gene-trait pairs retained.
The threshold implemented in study (L2G>0.5) shaded in red.

L2G threshold	Total	*ATXN2*	*HTT*	*ATXN1*	*CACNA1A*	*ATXN7*	*THAP11*	*ATXN3*	*ATN1*	*TBP*
0.0	3095	1063	466	259	469	86	260	178	235	79
0.1	854	541	101	108	15	59	1	22	2	5
0.2	570	293	87	104	14	54	0	14	1	3
0.3	400	149	81	94	13	47	0	14	0	2
0.4	306	98	68	80	11	35	0	12	0	2
**0.5**	**215**	**62**	**55**	**71**	**11**	**4**	**0**	**10**	**0**	**2**
0.6	122	25	21	58	10	0	0	7	0	1
0.7	69	0	9	48	10	0	0	2	0	0
0.8	10	0	1	0	9	0	0	0	0	0
0.9	0	0	0	0	0	0	0	0	0	0
1.0	0	0	0	0	0	0	0	0	0	0
